# Enhanced atmospheric water harvesting efficiency through green-synthesized MOF-801: a comparative study with solvothermal synthesis

**DOI:** 10.1038/s41598-023-44367-1

**Published:** 2023-10-09

**Authors:** Mohammad Aghajani Hashjin, Shadi Zarshad, Hosein Banna Motejadded Emrooz, Sadegh Sadeghzadeh

**Affiliations:** Nanotechnology Department, School of Advanced Technologies, Iran University of Science and Technology (IUST), Narmak, Tehran, 16846 Iran

**Keywords:** Organic-inorganic nanostructures, Design, synthesis and processing, Environmental impact

## Abstract

Adsorption-based atmospheric water harvesting has emerged as a compelling solution in response to growing global water demand. In this context, Metal–organic frameworks (MOFs) have garnered considerable interest due to their unique structure and intrinsic porosity. Here, MOF 801 was synthesized using two different methods: solvothermal and green room temperature synthesis. Comprehensive characterization indicated the formation of MOF-801 with high phase purity, small crystallite size, and excellent thermal stability. Nitrogen adsorption–desorption analysis revealed that green-synthesized MOF-801 possessed an 89% higher specific surface area than its solvothermal-synthesized counterpart. Both adsorbents required activation at a minimum temperature of 90 °C for optimal adsorption performance. Additionally, green-synthesized MOF-801 demonstrated superior adsorption performance compared to solvothermal-synthesized MOF-801, attributed to its small crystal size (around 66 nm), more hydrophilic functional groups, greater specific surface area (691.05 m^2^/g), and the possibility of having a higher quantity of defects. The maximum water adsorption capacity in green-synthesized MOF-801 was observed at 25 °C and 80% relative humidity, with a value of 41.1 g/100 g, a 12% improvement over the solvothermal-synthesized MOF-801. Remarkably, even at a 30% humidity level, green-synthesized MOF-801 displayed a considerable adsorption capacity of 31.5 g/100 g. Importantly, MOF-801 exhibited long-term effectiveness in multiple adsorption cycles without substantial efficiency decline.

## Introduction

Currently, freshwater scarcity stands as one of humanity's most pressing concerns. It is expected that water stress will worsen in the near future, particularly for individuals residing in dry areas^1,2^. Only a minor fraction, approximately 2.5%, of the global water supply is freshwater, while the overwhelming majority, around 97.5%, is saltwater in oceans. Out of the freshwater sources, only 0.4% is easily accessible and available for use^3,4^. Worldwide, research efforts have been taken to harvest water, including rainwater collection, groundwater harvesting, and water desalination. However, these methods heavily rely on the presence of liquid water sources and face significant limitations in regions that are landlocked^5,6^. It is a well-established fact that freshwater on Earth also includes water vapor present in the atmosphere, amounting to a total of 15 trillion liters^7,8^. Surprisingly, even in landlocked regions, a significant amount of water vapor exists. The atmospheric water generation technique not only presents labor and energy saving but also demonstrates its applicability across vast areas on Earth^9–12^. Furthermore, the harvested water by this method is suitable for drinking without the need for complex sterilization processes^7,13^. Typically, there are three main approaches to achieving atmospheric water generation: fog collection, cooling air to a temperature below the dew point, and employing sorbents to assist in water harvesting^14,15^. However, the first two methods face significant restrictions due to specific geographical or climatic conditions. As a result, considerable research efforts have been dedicated to investigating sorbent-assisted water capture^16,17^.

In recent years, there has been significant interest in a newly developed category of adsorbent materials known as "metal–organic frameworks (MOFs)"^18,19^. MOFs are a unique class of crystalline porous materials composed of metal ions or clusters connected by organic bridging ligands^20,21^. While MOFs have attracted considerable attention for various applications such as photocatalysis^22^, separation techniques^23,24^, electro-catalysis^25^, biomedical imaging^26^, drug delivery^27^, electronic and gas sensors^28,29^, researchers have recently begun exploring their potential as adsorbents for capturing water due to their exceptional porosity and expansive surface area^30^. MOFs designed for atmospheric water harvesting can be categorized into three main groups: (1) those based on aluminum, including MOF-303, CAU-10, and MIL-53; (2) zirconium-based MOFs, such as MOF-801, MOF-808, and MOF-841; and (3) other types like MIL-101 and Co_2_Cl_2_(BTDD)^31^. Among the MOFs, MOF-801, MOF-808, and MOF-841, stand out for their exceptional water adsorption capacity. They are notable for their ease of regeneration at temperatures below 150 °C. Even after five cycles of adsorption and desorption, these materials retained their water adsorption capabilities^32^. MOF-801 [${\mathrm{Zr}}_{6}{\mathrm{O}}_{4}{(\mathrm{OH})}_{4}{(\mathrm{fumarate})}_{6}]$ is a highly researched material in this category due to its several advantages such as its low cost and exceptional stability. Additionally, MOF-801 possesses three distinct cavities providing the ability to capture and concentrate water molecules within them^33–36^. It has been previously reported that MOF-801 exhibits excellent water adsorption performance even in regions with low humidity^4^. Kim et al. demonstrated a remarkable water adsorption capacity of ~ 25 g/100 g by utilizing MOF-801 at a low relative humidity of 20%^37^. In another study, Yaghi et al. demonstrated a maximum adsorption capacity of 22.5 g/100 g for MOF-801 at 10% RH^18^. The same research group designed a water harvesting device based on MOF-801, which is capable of collecting 2.8 L of water per kilogram of adsorbent per day at 20% RH without requiring any additional energy input^38^. In another study, Mi et al. suggested a way for synthesizing defective MOF-801 which results in an exceptional level of water harvesting efficiency (1.60 kg water/kg adsorbent/day at 20% RH)^39^.

Despite the numerous studies conducted on the application of Zr-based MOFs for water harvesting, a comprehensive investigation that thoroughly assesses the effect of surface characteristics and environmental parameters such as temperature and humidity level on the water harvesting performance of MOF-801 is noticeably lacking. Moreover, there is a scarcity of research that specifically examines the optimum activation temperature and water adsorption rate of MOF-801. Additionally, existing research in this domain have not sufficiently explored the morphology, structure, and adsorption performance of MOF-801 after undergoing multiple and prolonged cycles. To overcome the mentioned lack of information, in this study, MOF-801 was synthesized as a water adsorbent using two different methods: (1) solvothermal method with DMF as the solvent, and (2) room temperature green-synthesis with water as the solvent. The synthesized samples were compared in terms of their characteristics, morphology, and structure. Subsequently, the optimum activation temperature of MOF-801 was investigated to determine the temperature at which all adsorbed water is desorbed, resulting in complete water removal. The performance of the adsorbents was then examined under various temperature and humidity conditions. Additionally, the adsorption rate in MOF-801 was studied to assess its performance within a specific time frame. Finally, the characteristics, adsorption performance, and stability of MOF-801 over multiple adsorption cycles were discussed, to evaluate its efficiency for repeated use.

## Experimental section

### Materials

Fumaric acid (${\mathrm{C}}_{4}{\mathrm{H}}_{4}{\mathrm{O}}_{4}$, purity ≥ 99.0%), Zirconium oxychloride octahydrate (ZrO ${\mathrm{Cl}}_{2}$·8 ${\mathrm{H}}_{2}$ O, purity ≥ 99.5%), Formic acid (HCOOH, purity ≥ 98.0%), *N,N’*-dimethylformamide (DMF, HPLC grade), Ethanol (purity ≥ 99.0%), Methanol (HPLC grade), and Sulfuric acid (purity: 95–97%) all from Sigma-Aldrich Co, and Deionized water. It is noteworthy that all the materials were used without any additional treatment.

### Solvothermal-synthesized MOF-801

In the first method (solvothermal synthesis), MOF-801 was synthesized according to the synthesis procedure described by Yaghi and co-workers^40^ as follows: Firstly, in a screw-capped jar, 1.24 g fumaric acid and 3.42 g ZrO ${\mathrm{Cl}}_{2}$·8 ${\mathrm{H}}_{2}$ O were dissolved in a solvent mixture consisting of 42.8 mL of DMF and 14.98 mL of formic acid under magnetic stirring. The resulting solution was then transferred into an autoclave and heated at 130 °C for 10 h. The white product was filtrated and washed three times with 50 mL of DMF over a period of 3 days. The washed particles were dried overnight at ambient temperature, followed by another round of three daily washes with 50 mL of methanol for 3 days and subsequently dried in air again. Finally activated MOF-801 was obtained by drying the white solid at 150 °C under vacuum for 24 h. For the sake of naming convenience, during this study, the solvothermal-synthesized MOF-801 is named SS-MOF-801.

### Green-synthesized MOF-801

The second method (green room temperature synthesis) used water instead of DMF as the solvent to synthesize MOF-801 according to the previous report^41,42^. In this case, 3.5 g ZrO ${\mathrm{Cl}}_{2}$·8 ${\mathrm{H}}_{2}$ O was dissolved in a distilled water/formic acid mixed solvent (80 mL/20 mL). After 1 min of stirring, 1.75 g of fumaric acid was added and stirred at 600 rpm for 90 min to form a homogeneous solution. The resulting solution was left at room temperature for 48 h to produce a cloudy solution, indicating the formation of MOF-801. Afterward, the white precipitate was collected by centrifugation, followed by washing one time with water and two times with ethanol, placing at room temperature for 48 h, and finally drying at 150 °C under vacuum for 7 h. The green-synthesized MOF-801 is named GS-MOF-801 during this study.

### Characterization

The crystallinity of the MOF-801 was carried out by X-ray diffraction (XRD, PW 1730 diffractometer, PHILIPS, Netherlands) in the 2θ range from 5° to 80° with a step of 0.04° using CuKα radiation (λ = 1.5417 Å). The instrument's settings included a voltage of 40 kV and a current of 30 mA. The functional groups and chemical bonds of synthesized MOF were analyzed by Fourier transform infrared spectroscopy (FTIR, Spectrum RX1, PERKIN-ELMER, USA) over the scanning range of 400 to 4000 cm^−1^ wavenumbers with a spectral resolution of 1 cm^-1^. The thermal behavior of MOF-801 was investigated using a Thermogravimetric analyzer (TGA, STA504, BAHR, Germany) over a temperature range of 50 to 1000 °C, using a heating rate of 10 °C min^−1^, under a nitrogen atmosphere. The specific surface area and porosity of the samples were evaluated from the nitrogen adsorption–desorption isotherms at 77 K (BELSORP-mini II, Bel Inc., Japan). The morphology and elemental composition of MOF-801 crystals were analyzed by field emission scanning electron microscopy (FESEM, EM8000, KYKY, China). A Transmission electron microscope (TEM, CM120, PHILIPS, Netherlands) was employed to investigate the structure and crystal morphology of the samples.

### Water adsorption test

**Figure 1:**
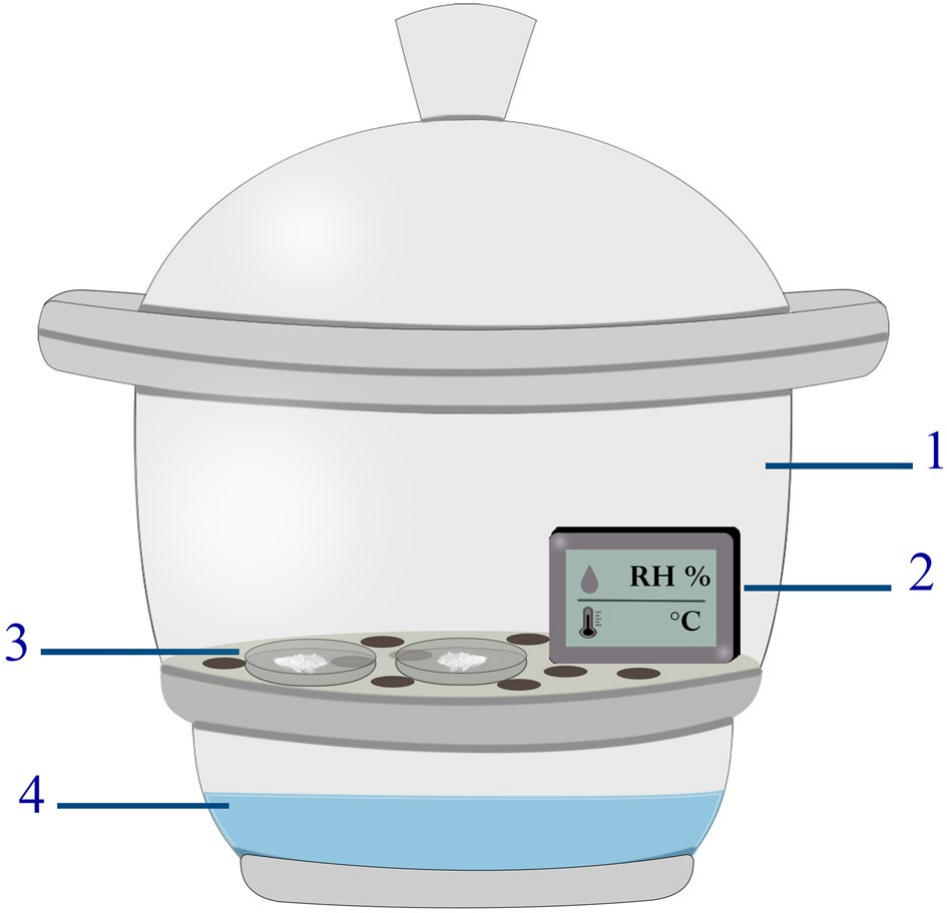
Schematic of the equipment for determination of adsorption performance of MOF-801: (1) desiccator, (2) digital temperature humidity meter, (3) sample in the Petri dish, (4) sulfuric acid solution.

To set up a constant temperature and humidity environment, a desiccator filled with different concentrations of sulfuric acid solution was used. To this end, sulfuric acid was diluted with deionized water to make specific relative humanity inside the desiccator. Besides, for monitoring the temperature and humidity, a digital temperature humidity meter was placed in the desiccator. A schematic of the set-up for determination the adsorption performance is presented in Fig. 1. As a first step, the samples were placed in an oven to remove the adsorbed water vapor before the adsorption test. After the desorption process, dried samples were weighed and placed in a Petri dish inside the desiccator and allowed to adsorb water vapor from the moisture-controlled environment. The weight increase method was utilized to measure the adsorption capacity of the adsorbent which is expressed as the water uptake per unit weight of adsorbent and with unit g/100 g. The water uptake of samples is given^43^ by the following equation:

1 $$\mathrm{Water \, uptake }= \frac{{\mathrm{m}}_{\mathrm{ad}}-{\mathrm{m}}_{\mathrm{de}}}{{\mathrm{m}}_{\mathrm{de}}}$$

where ${\mathrm{m}}_{\mathrm{ad}}$ is the mass of the MOF-801 after the water adsorption test and ${\mathrm{m}}_{\mathrm{de}}$ is the mass of the MOF-801 after desorption by heating. This method was employed for multiple sets of conditions in terms of temperature and humidity to determine the adsorption performance of MOF-801. All adsorption tests in this study were conducted over 24 h. “The maximum adsorption capacity” for each sample, which represents the adsorption capacity of the adsorbent at the end of the 24-h adsorption process, was compared with the others.

### Activation temperature

The activation temperature plays a crucial role in the efficiency of an adsorbent. It signifies the temperature at which the adsorbed water within the adsorbent structure is completely eliminated. To investigate the optimal activation temperature, the synthesized samples that had been exposed to 80% (± 5%) RH for 2 h, were subjected to different temperature conditions (70 °C, 90 °C and 110 °C) to remove the adsorbed water from the adsorbents. Subsequently, the mass changes of the samples were measured after a 1-h desorption process. Following that, the adsorption test was carried out under the conditions of 25 °C ± 1 °C and 80% (± 5%) RH. Finally, the adsorption performance of samples during the 24-h adsorption process was compared. Each sample was labeled with the corresponding activation temperature of the adsorbent. For instance, SS-MOF-801-70 refers to SS-MOF-801 that has undergone a heating process at 70 °C in order to eliminate the water adsorbed within it.

## Results and discussion

### Characterization

**Figure 2:**
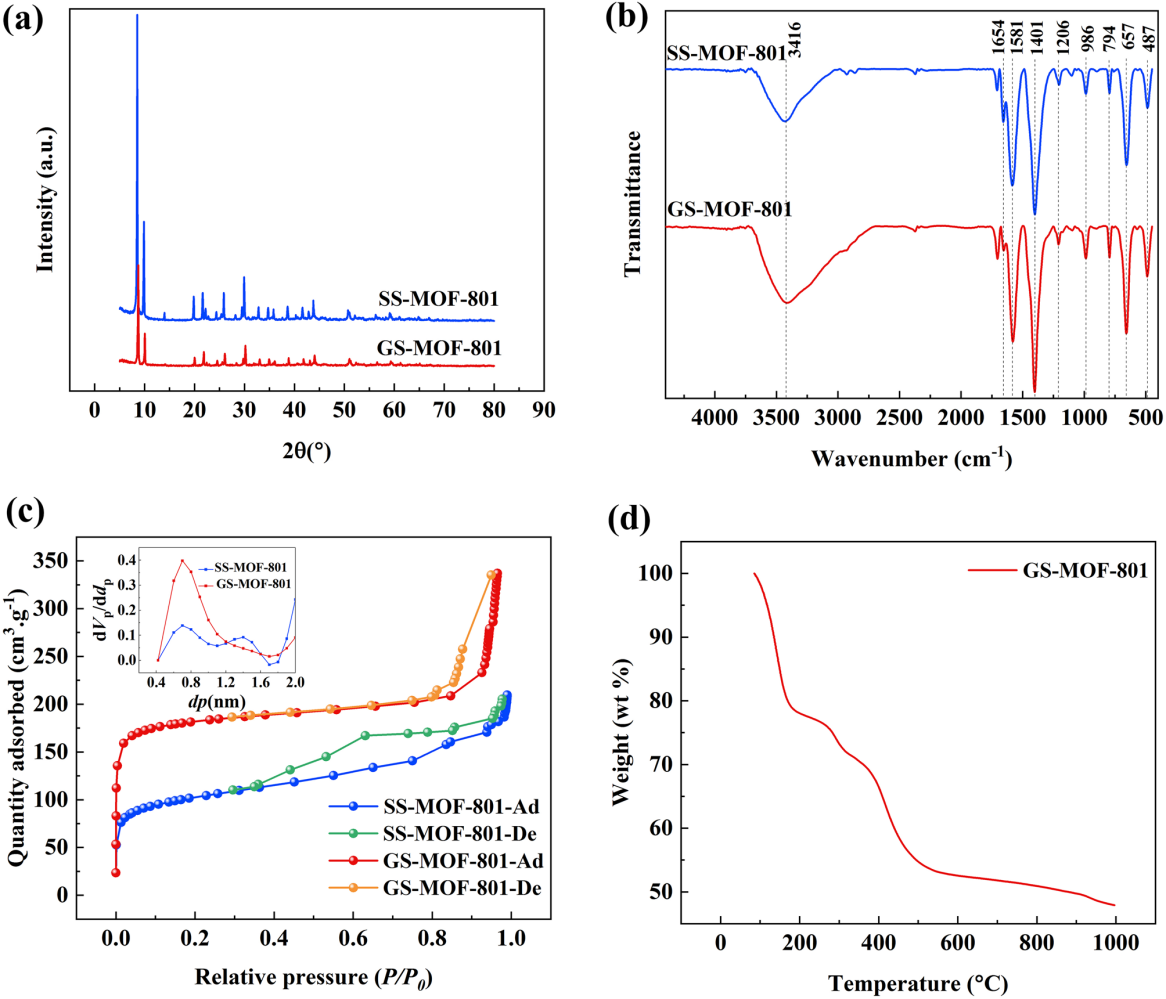
XRD patterns (**a**), FTIR spectra (**b**), and the nitrogen adsorption at 77 K and pore size distribution curve (inset) (**c**) of SS-MOF-801 and GS-MOF-801; TGA curve of GS-MOF-801 (**d**).

The XRD patterns of both the SS-MOF-801 and GS-MOF-801 are depicted in Fig. 2a. It can be seen that both samples exhibit distinct peaks at angles of 8.5° and 9.8°. These peaks correspond to the crystal faces of MOF-801, specifically the (1 1 1) and (2 0 0) planes, respectively. This result is in good agreement with the XRD patterns of previous studies^44,45^ indicating the high phase purity of the powder samples. The crystallite size of the SS-MOF-801 and GS-MOF-801 is ~ 46 nm and ~ 66 nm respectively. According to Grosu’s report, small crystallite size can lead to a significant decrease in intrusion and extrusion pressures, which can be an advantage for water harvesting systems^46^. From the FTIR spectrum of the synthesized SS-MOF-801 and GS-MOF-801 shown in Fig. 2b, a broad peak around 3416 cm^−1^ indicates the presence of the OH group^47^. The peaks at 1654 cm^−1^,1581 cm^−1^, and 1401 cm^−1^ could be attributed to –C=O–O bonds^48^. On the other hand, the peaks at 1206 cm^−1^, 986 cm^−1^, and 794 cm^−1^ are identified as the C–H vibrations^49^. Finally, the absorption at 657 cm^−1^ and 487 cm^−1^ could be assigned to ${\mathrm{Zr}}_{6}{(\mathrm{OH})}_{4}{\mathrm{O}}_{4}$ vibrations and Zr-(OC) asymmetric stretching, respectively^50^. As can be seen, the increase in the OH peak area of GS-MOF-801 can signify the sample contains more hydrophilic functional groups compared to SS-MOF-801. And there may be more opportunities for intermolecular interactions and hydrogen bonding, which could lead to a better water adsorption behavior of GS-MOF-801. Consequently, the utilization of water in the synthesis of MOF-801 not only addresses the drawback of employing harmful solvents like DMF but also leads to the formation of more hydrophilic functional groups, which offers benefits for water adsorption.

The graph in Fig. 2c displays the nitrogen adsorption–desorption isotherms and micropore size distribution of the powder samples of MOF-801, which are related to Type-I adsorption isotherm^51^. It should be highlighted that a slight inflexion of the adsorption isotherm curve in both samples, can be related to the occurrence of some defects^52^. Besides, according to Li's report, the enhanced nitrogen uptake during the physisorption of GS-MOF-801 can be attributed to the presence of the highest defects in its framework compared to the SS-MOF-801. The mentioned defects within the crystal lattices provide numerous adsorption sites for water. Additionally, the concentration of defects in the GS-MOF-801 can be adjusted by varying the amount of the modulator (formic acid) used in the aqueous synthesis. It is important to mention that the amount of formic acid employed in the present work was determined as the optimal quantity based on the previous study to achieve the highest possible defect concentration in GS-MOF-801^39,42^. The specific surface area of SS-MOF-801 and GS-MOF-801 is 365.43 m^2^/g and 691.05 m^2^/g respectively. On the other hand, SS-MOF-801 shows two distinct types of micropores, which have radii of 0.7 nm and 1.4 nm. In contrast, GS-MOF-801 has a main pore size, located at a radius of 0.7 nm. GS-MOF-801 is expected to outperform SS-MOF-801 in terms of water adsorption. This expectation is based on the potential for GS-MOF-801 to possess a greater quantity of defects and an 89% larger specific surface area, enabling more active sites for water molecules to interact with and be adsorbed. Figure 2d shows the TGA curve of synthesized GS-MOF-801 crystals. A 23% weight loss occurs below ~ 200 °C, owing to the removal of adsorbed water and guest molecules trapped within the adsorbent's pores. Subsequently, the sharp declining trend starts to begin above 350 °C and extends up to 500 °C which is attributed to the loss of fumaric acid and breaking of the carboxylate groups. This observation points to the adsorbent's excellent thermal stability^42,51^.

**Figure 3:**
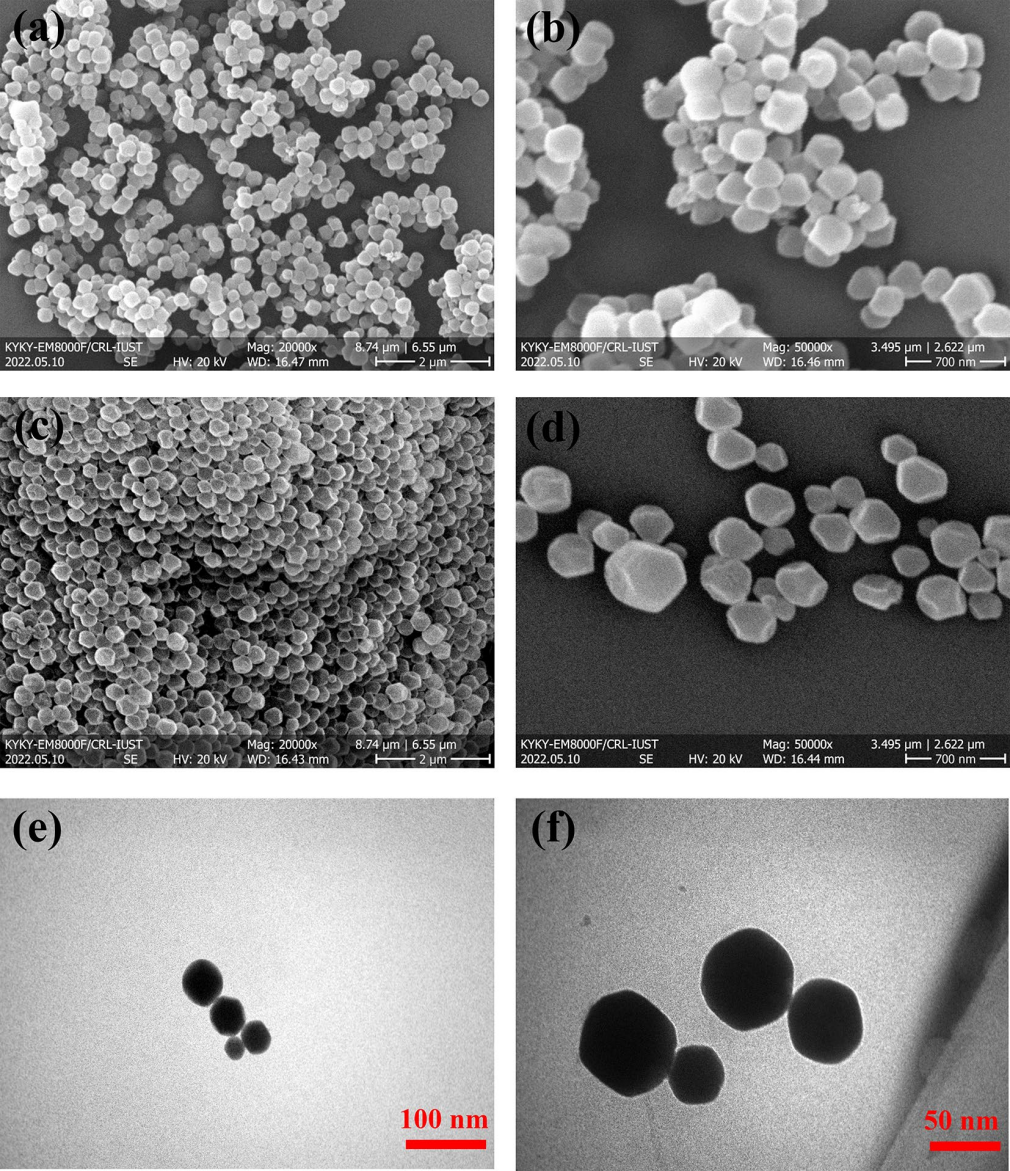
FESEM images of SS-MOF-801 (**a**, **b**) and GS-MOF-801 (**c**, **d**); TEM images of GS-MOF-801 (**e**, **f**).

The morphology and size of the synthesized samples were subsequently analyzed using FESEM and TEM. The FESEM micrographs demonstrate that both SS-MOF-801 and GS-MOF-801 have a uniform dispersion and size distribution (Fig. 3a–d). Nevertheless, SS-MOF-801 displays an octahedral structure, whereas GS-MOF-801 tends to crystallize in a rounded form. These findings align with previous research indicating that the water-based synthesis of MOF-801 predominantly yields spherical particles instead of octahedral ones^53^. On the other hand, based on the FESEM (Fig. 3a–d) and TEM (Fig. 3e,f) images, it can be observed that the particle size of both MOF-801 samples is less than 200 nm, which is in accordance with previously reported values^54^.

### Activation temperature

**Figure 4:**
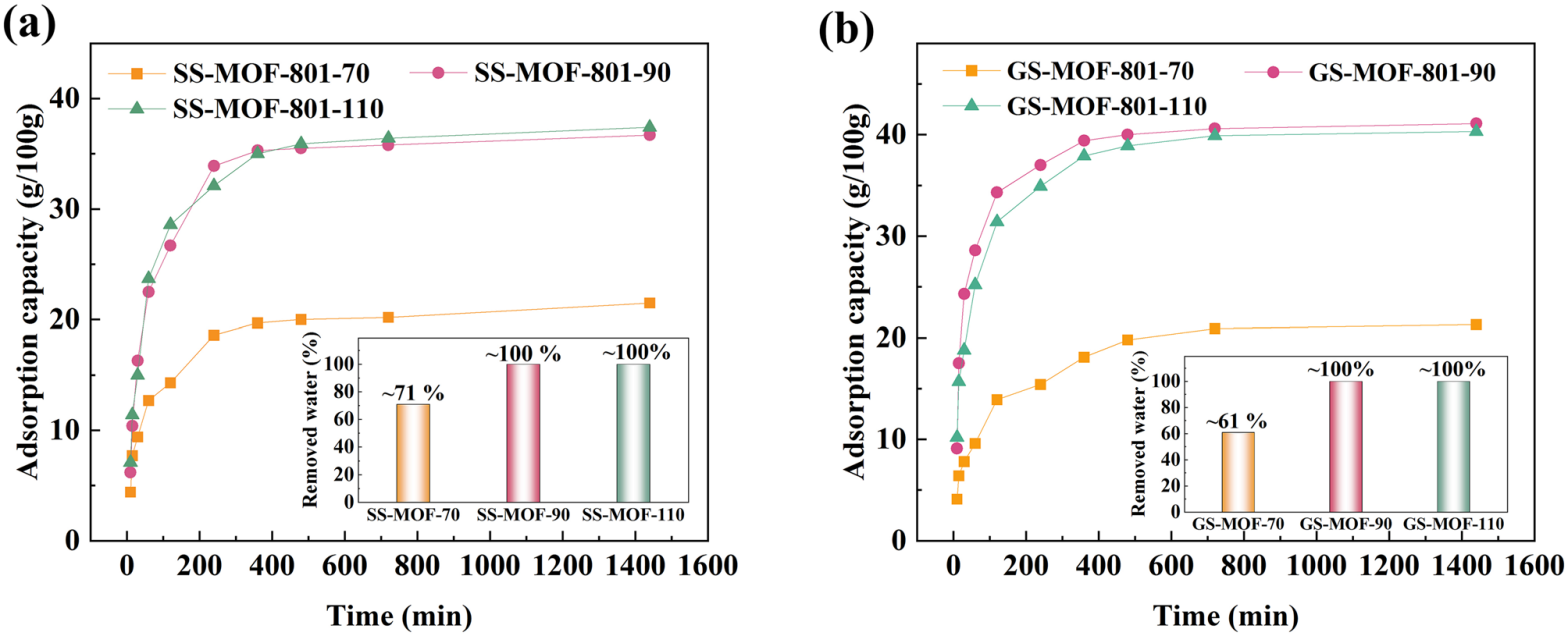
Water sorption behavior of SS-MOF-801 (**a**) and GS-MOF-801 (**b**) with time; Inset: the amount of water removed from the total adsorbed water in corresponding samples after a 1-h desorption process.

It can be seen from Fig. 4 that the adsorption performance of the adsorbents varies depending on the activation temperature. In Fig. 4a, it can be observed that SS-MOF-801-90 achieves a maximum adsorption capacity of 36.7 g/100 g at the end of the 24-h adsorption process. This value represents a 71% increase compared to the adsorption capacity of SS-MOF-801-70, which is 21.5 g/100 g. This significant difference can be attributed to the fact that a temperature of 70 °C is inadequate for the complete removal of adsorbed water from the adsorbent cavities and preparing the adsorption sites sufficiently for the adsorption process^42,44^. Additionally, as observed in Fig. 4b, similar findings are observed for GS-MOF-801-90 too, and its maximum adsorption capacity exceeds that of GS-MOF-801-70 by 92%. Furthermore, the inset of Fig. 4a and b displays the amount of water removed from the total adsorbed water in corresponding samples at the end of the desorption process. It can be seen that the adsorbed water content in MOF-801-90 and MOF-801-110 has been completely removed during the desorption process, whereas in the case of SS-MOF-801-70 and GS-MOF-801-70, it has been reduced by 71% and 61% respectively. In earlier studies, it has been indicated that MOF-801 demonstrates a lower activation temperature in comparison to other adsorbents^55,56^, and the current observation confirms that the optimum activation temperature for both SS-MOF-801 and GS-MOF-801 adsorbents is 90 °C. It is obvious that the impact of the activation temperature of 110 °C on the water adsorption performance of both samples is also favorable. It is worth mentioning that the maximum adsorption capacity of GS-MOF-801-90 is 41.1 g/100 g, which represents a 12% increase compared to SS-MOF-801-90. This difference can be attributed to GS-MOF-801's significantly larger specific surface area, which is 89% greater than that of SS-MOF-801. Additionally, the potential for a higher quantity of defects and the presence of more hydrophilic functional groups in GS-MOF-801 compared to SS-MOF-801 may further contribute to this difference. Therefore, alongside the use of water instead of the harmful DMF as the solvent for the synthesis of GS-MOF-801, it exhibits superior water adsorption performance in comparison to SS-MOF-801.

### The effect of temperature on water sorption

**Figure 5:**
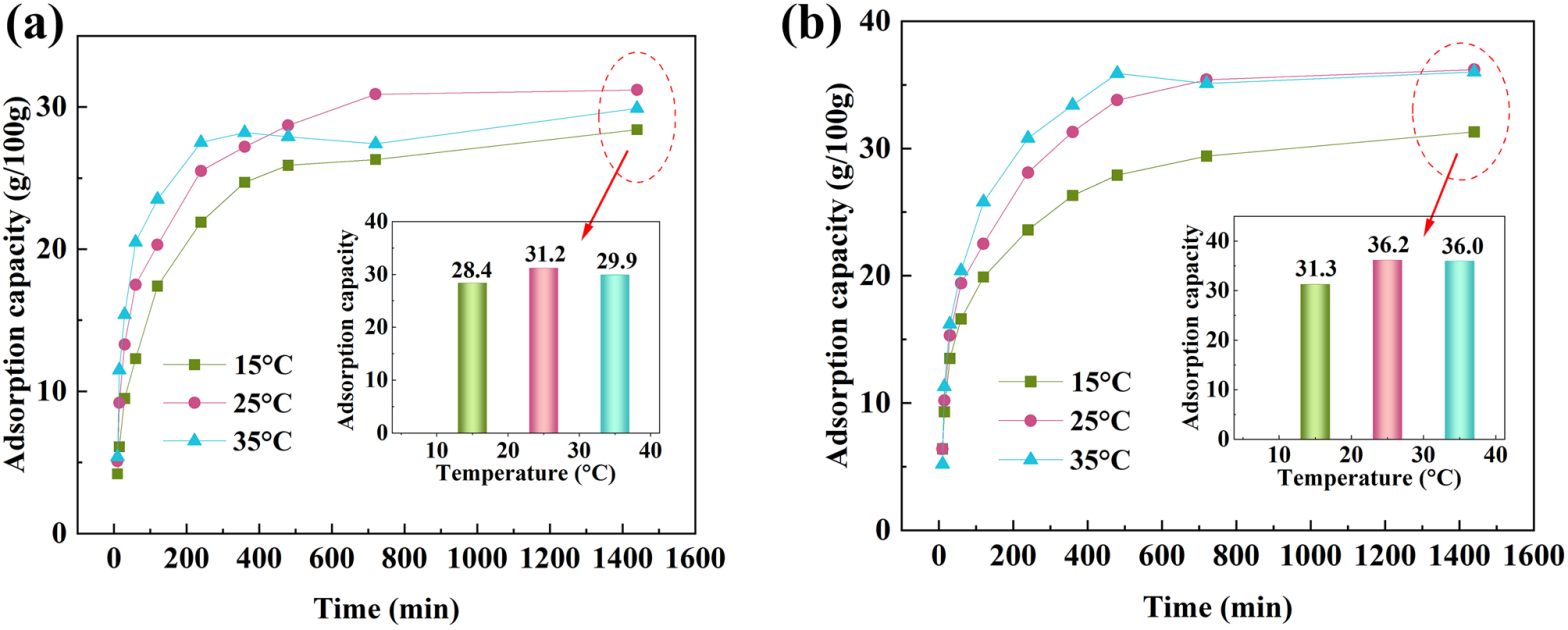
Water sorption behavior of SS-MOF-801 (**a**) and GS-MOF-801 (**b**) at different temperatures.

In order to investigate the influence of temperature on the adsorption performance of the synthesized adsorbents, the samples were first activated at the temperature of 90 °C. Subsequently, adsorption tests were conducted under a controlled relative humidity of 50% (± 5%) at different temperatures: 15 °C, 25 °C, and 35 °C (± 1 °C). The results are depicted in Fig. 5. As observed in Fig. 5a, the maximum adsorption capacity of SS-MOF-801 at 25 °C is 31.2 g/100 g, but it decreases to 28.4 g/100 g when the temperature drops to 15 °C. In other words, the maximum adsorption capacity of SS-MOF-801 at 15 °C is 9% lower than that at 25 °C. Overall, at a constant relative humidity of 50%, SS-MOF-801 demonstrates its optimum adsorption performance at a temperature of 25 °C. When the temperature is 35 °C, the maximum adsorption capacity of SS-MOF-801 decreases by 4% compared to that at 25 °C. Furthermore, as depicted in Fig. 5, the water adsorption curves of MOF-801 at 35 °C exhibit signs of instability after a duration of 6 h. The findings suggested that elevated temperatures can lead to a slight reduction in the water adsorption capacity of adsorbents under the condition of constant relative humidity. A similar behavior was also previously observed in the MIL-101(Cr) adsorbent^57^. This can be attributed to the activation of certain water molecules at higher temperatures, causing them to detach from the sorption sites within the adsorbents. Consequently, this results in a decline in the adsorption efficiency of the samples^58^. According to Fig. 5b, the highest adsorption capacity of GS-MOF-801 is also observed at 25 °C, reaching 36.2 g/100 g. This value is 16% higher than that of SS-MOF-801.

### The effect of relative humidity on water sorption

**Figure 6:**
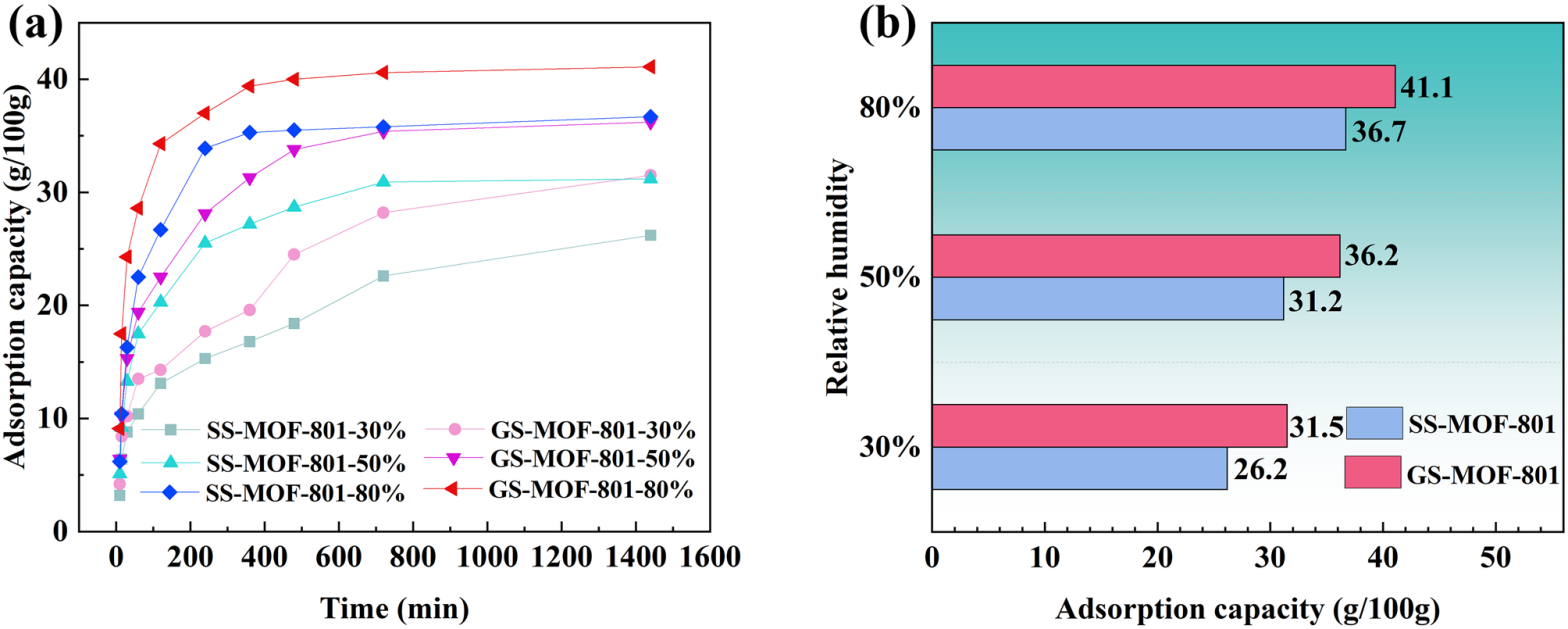
Water Sorption Behavior of SS-MOF-801 and GS-MOF-801 at different relative humidity (**a**); The maximum adsorption capacity of SS-MOF-801 and GS-MOF-801 at various relative humidity and constant temperature of 25 °C (**b**).

It is expected that humidity would significantly impact the water adsorption performance of an adsorbent. In this regard, the water adsorption performance of synthesized adsorbents was investigated at a constant temperature of 25 °C (± 1 °C), under various relative humidity conditions of 30%, 50%, and 80% (± 5%) (Fig. 6). As seen in Fig. 6, both SS-MOF-801 and GS-MOF-801 exhibit an increase in adsorption performance with increasing humidity. Specifically, the maximum adsorption capacity of SS-MOF-801 and GS-MOF-801 increases by 19% and 15%, respectively, when the humidity rises from 30 to 50%. Furthermore, with an increase in humidity from 50 to 80%, the maximum adsorption capacity of the mentioned adsorbents also increases by 17% and 13% respectively. Naturally, this phenomenon can be attributed to the fact that as the relative humidity increases, the pressure of water vapor also increases at a constant temperature leading to better water adsorption in adsorbents^57^. Furthermore, as observed in Fig. 6, GS-MOF-801 exhibits better performance in water adsorption compared to SS-MOF-801 at all humidity levels. It should be noted that even at a relative humidity of 30%, GS-MOF-801 demonstrates a considerable adsorption capacity of 31.5 g/100 g. This indicates that MOF-801 can also be effective in low humidity conditions (e.g., in North Africa).

### Adsorption rate of adsorbents

The adsorption rate during the initial hours plays a pivotal role in determining the efficiency of water intake when employing adsorbents for this purpose^57,59^. One crucial observation that can be seen in all water adsorption curves of MOF-801 is the high adsorption rate of adsorbents in the initial four hours of the adsorption process. As a result, the majority of water adsorption occurs within the mentioned time frame. For example, when considering GS-MOF-801 under the conditions of 25 °C and 50% RH (Fig. 6a), ~ 78% of the maximum adsorption capacity is achieved within the first four hours. Similarly, at a temperature of 25 °C and relative humidity of 80% (Fig. 6a), this value increases to ~ 90%. Therefore, in order to examine the adsorption rates of SS-MOF-801 and GS-MOF-801 under different temperature and humidity conditions, the adsorption rate during the first four hours, referred to as " Adsorption rate I" was compared with the adsorption rate during the subsequent four hours, referred to as " Adsorption rate II". The results can be observed in Tables 1 and 2. Based on the findings presented in Tables 1 and 2, it is apparent that the adsorbents exhibit a noticeably higher rate of water adsorption during the first four hours compared to the subsequent four hours. Hence, in situations where there is a time limitation for utilizing the adsorbent for water adsorption, MOF-801 can be considered as a suitable option since it proves to exhibit a substantial level of adsorption within the initial hours of the adsorption process. Furthermore, the highest adsorption rate is observed in GS-MOF-801 at a temperature of 25 °C and relative humidity of 80% RH. Moreover, it can be seen that as the temperature and relative humidity levels rise, the water adsorption rate I also increases. From Table 2, it can be concluded that the cavities of adsorbents become saturated more quickly in high humidity levels compared to low humidity levels. As a result, the adsorption rate II decreases as the humidity level increases.

**Table 1:** Maximum adsorption capacity and adsorption rate of SS-MOF-801 and GS-MOF-801 at 50% RH.

Sample	Temperature (°C)	Maximum adsorption capacity (g/100 g)	Adsorption rate I (g kg^−1^ h^−1^)	Adsorption rate II (g kg^−1^ h^−1^${\mathrm{h}}^{-1}$)
SS-MOF-801	15	28.4	54.7	10.0
	25	31.2	63.7	8.0
	35	29.9	68.7	1.0
GS-MOF-801	15	31.3	59.0	10.7
	25	36.2	70.2	14.2
	35	36.0	77.0	12.7

**Table 2:** Maximum adsorption capacity and adsorption rate of SS-MOF-801 and GS-MOF-801 at 25 °C.

Sample	RH (%)	Maximum adsorption capacity (g/100 g)	Adsorption rate I (g kg^−1^ h^−1^)	Adsorption rate II (g kg^−1^ h^−1^)
SS-MOF-801	30	26.2	38.2	7.7
	50	31.2	63.7	8.0
	80	36.7	84.7	4.0
GS-MOF-801	30	31.5	44.2	17.0
	50	36.2	70.2	14.2
	80	41.1	92.5	7.5

### Reuse performance of adsorbent

**Figure 7:**
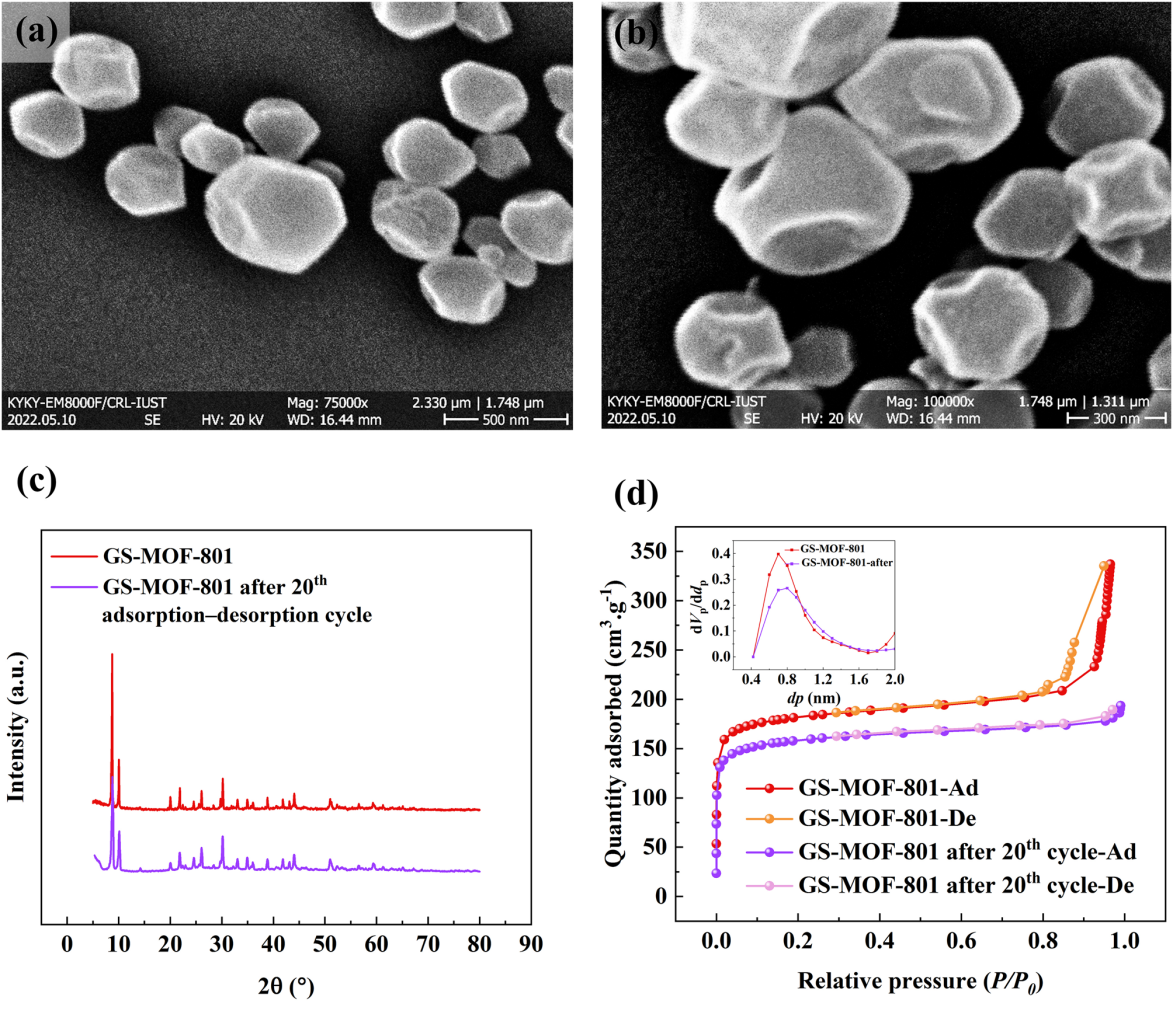
FESEM images of GS-MOF-801 before the first adsorption cycle (**a**) and after the 20th adsorption–desorption cycle (**b**); XRD pattern (**c**) and the nitrogen physisorption at 77 K and pore size distribution curve (inset) (**d**) of GS-MOF-801 during adsorption–desorption cycling test.

It is crucial for an appropriate adsorbent to sustain its efficiency over consecutive sorption cycles without experiencing a significant performance decline^32^. Hence, this aspect will be thoroughly examined in this section. Furthermore, as GS-MOF-801 exhibits superior adsorption performance in comparison to SS-MOF-801, the characteristics and performance of GS-MOF-801 will be discussed over multiple cycles. It is worth mentioning that each cycle lasted 24 h, and the cycles were carried out under varying conditions of temperature and humidity levels. For this purpose, after each adsorption cycle, GS-MOF-801 underwent a desorption process at a temperature of 90 °C to remove the adsorbed water, and it was subsequently used for the next adsorption cycle. Figure 7a and b depict the FESEM images of GS-MOF-801 before the first cycle and after the 20th adsorption–desorption cycle, respectively. The FESEM images show that the GS-MOF-801 particles exhibit a sign of slight deformation compared to their initial shape after undergoing consecutive adsorption–desorption cycles. Besides, based on the observations in Fig. 7c, it is evident that even after undergoing multiple cycles, the crystallinity of GS-MOF-801 remains unaffected. The graph in Fig. 7d and its inset demonstrate the impact of 20 cycles of water adsorption–desorption on nitrogen physisorption and pore size distribution of GS-MOF-801. As can be seen, after consecutive adsorption cycles, the pore size of GS-MOF-801 remained relatively stable with no significant changes. And the specific surface area of the adsorbent decreased to 600.35 m^2^/g after 20 cycles, representing a 13% reduction compared to its initial value which could be attributed to factors such as slight structural degradation or exhaustion of the adsorbent. Nevertheless, this decrease can be considered negligible. As a consequence, the aforementioned results indicate that MOF-801 exhibits excellent stability over multiple cycles.

**Figure 8:**
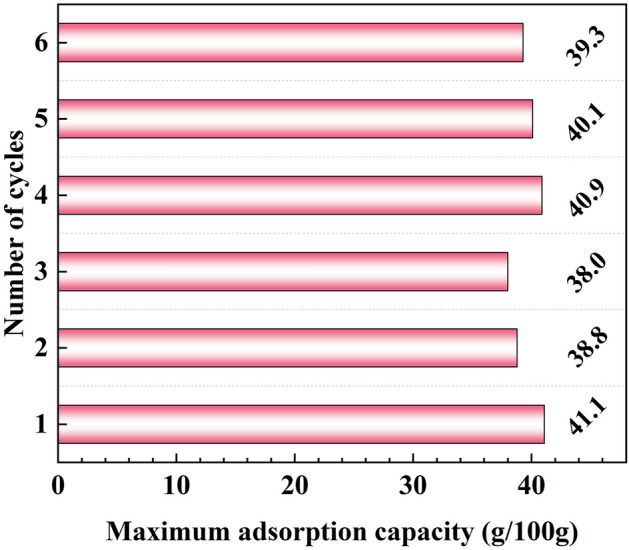
The water adsorption performance of GS-MOF-801 during adsorption–desorption cycling test.

In order to investigate the recycling performance of the adsorbent, the adsorption performance of GS-MOF-801 was examined over 6 cycles at a temperature of 25 °C (± 1 °C) and 80% (± 5%) RH. This evaluation aimed to assess whether MOF-801 maintains its water adsorption properties consistently and remains durable and reliable over extended periods of use. For this purpose, after each adsorption test under the mentioned temperature and humidity conditions, the adsorbent was heated to 90 °C to remove the adsorbed water before the next cycle and then tested again under similar conditions. The maximum adsorption capacity of GS-MOF-801 at the end of the 24-h adsorption process, for each cycle is shown in Fig. 8. As observed, the adsorption performance of GS-MOF-801 remains relatively constant throughout the 6 cycles, with ~ 39.5 g/100 g of the adsorption capacity remaining. Therefore, the adsorbent demonstrates high-performance stability during the 6 cycles. It should be noted that the slight increase observed in the fourth and fifth cycles, could be attributed to improved removal of water inside the pores during the pre-cycle desorption process. Furthermore, the absence of a significant decrease in adsorption capacity throughout the cycles indicates that the adsorbent has not suffered from any exhaustion or structural degradation over the 6 cycles.

## Conclusion

In this study, we investigated the water adsorption performance of MOF-801, focusing on factors such as hydrophilic functional groups, specific surface area of the adsorbent, and environmental conditions. MOF-801 was synthesized using two different methods, solvothermal and green synthesis. The green-synthesized MOF-801 outperformed solvothermal-synthesized MOF-801, attributed to several factors including its small crystallite size, increased presence of hydrophilic functional groups, larger specific surface area, and the possibility of having a higher quantity of defects. Furthermore, both adsorbents need to be activated at a minimum temperature of 90 °C to exhibit their best adsorption performance. The maximum adsorption capacity for MOF-801 was found under the conditions of 25 °C and 80% RH. MOF-801 also exhibited a considerable adsorption capacity even under low humidity conditions. Additionally, the high adsorption rate of MOF-801 during the initial hours highlights its suitability for applications where time constraint is a concern. Finally, the adsorption–desorption cycling tests revealed the long-term effectiveness of MOF-801 in multiple water adsorption cycles, with no significant decline in its efficiency. Overall, these findings demonstrate the potential of MOF-801 as a promising water adsorbent with excellent stability and enhanced adsorption performance, making it suitable for water harvesting even at low humidity conditions.

## Data Availability

All data presented in this study are included in this published article.
